# Monitoring of physical activity promotion in children and adolescents in the EU: current status and future perspectives

**DOI:** 10.1093/eurpub/ckab193

**Published:** 2021-11-17

**Authors:** Isabel Marzi, Antonina Tcymbal, Peter Gelius, Karim Abu-Omar, Anne K Reimers, Stephen Whiting, Kremlin Wickramasinghe

**Affiliations:** 1 Department of Sport Science and Sport, Friedrich-Alexander-University Erlangen-Nuremberg, Erlangen, Germany; 2 European Office for the Prevention and Control of Noncommunicable Diseases, Regional Office for Europe, World Health Organization, Moscow, Russian Federation; 3 EPIUnit—Instituto de Saúde Pública, Universidade do Porto, Porto, Portugal

## Abstract

**Background:**

Policy action is required to address physical inactivity in boys and girls. This action can be supported by international data collection, comparisons and sharing of good practices. Thus, this study aims to present and discuss the ongoing monitoring of physical activity (PA) indicators in children and adolescents in the 28 EU Member States.

**Methods:**

Data on PA recommendations, PA prevalence, physical education (PE) and PA promotion programs for children and adolescents were provided by governments in a joint EU/WHO survey on the implementation status of the EU Council Recommendation on Health-Enhancing Physical Activity (HEPA) across Sectors.

**Results:**

In 23 countries, national recommendations on PA are available. Detailed PA prevalence data among children and adolescents was available in 27 countries, in most cases separately for sex/gender and age groups. The total amount of PE lessons in schools differed greatly between countries and lessons were predominantly mandatory. After-school HEPA promotion programs were mostly implemented in EU Member States (78.6%), followed by active school breaks (57.1%), active travel to school (57.1%) and active breaks during school lessons (53.6%).

**Conclusions:**

This study summarizes the monitoring of PA indicators among children and adolescents in all EU Member States by providing a comprehensive overview of the status of PA promotion and monitoring across the region. Based on our findings, it could be concluded that the current EU monitoring system on PA promotion should be adapted to provide evidence that can inform future policy development.

## Introduction

Physical inactivity is one of the main risk factors for noncommunicable diseases,^1^ and benefits of physical activity (PA) for children’s and adolescents’ health,^2^^,^^3^ regardless of sedentary time,^4^ are well-known. Both cardiorespiratory and musculoskeletal fitness can be improved by increasing PA in children and adolescents.^2^^,^^3^ PA also positively affects body composition, i.e. higher levels of PA are associated with a healthy weight status in children and adolescents.^3^ In addition, regular PA is beneficial for academic achievement in children and adolescents.^5^

Despite these known benefits, <20% of 11- to 17-year-olds meet the recommendation provided by the World Health Organization of 60 min moderate to vigorous-intensity PA daily.^6^ Similar prevalence rates are reported in younger children.^7^ From 2001 to 2016, in spite of intensified research as well as the development and implementation of a growing number of interventions to promote PA, the prevalence of insufficient PA remained constant among girls, and only slightly decreased among boys.^6^

National and global action is required to achieve the global target of a 15% reduction in insufficient PA by 2030.^8^ As stated in the ‘Global Action Plan on PA 2018–30^’^, such action should consist of both standardized PA monitoring and evidence-based effective PA promotion programs. Furthermore, international data collection, comparisons and sharing of good practices are essential to reduce global inactivity prevalence. Such action should consist of both standardized PA monitoring and evidence-based effective PA promotion programs. Furthermore, international data collection, comparisons and sharing of good practices might help reduce global inactivity prevalence.

In the European Union (EU), policy actions to address physical inactivity are increasing. However, additional efforts in support of the collection, interpretation, and harmonization of data on the current PA monitoring and promotion across EU Member States could help to advance policy development.^9^ In EU Member States, data on health behaviors, such as PA among children and adolescents, are partly assessed within the Health Behavior in School-aged Children (HBSC) Study^10^ and the Childhood Obesity Surveillance Initiative.^11^ Such initiatives are important in that they enable comparisons between participating countries and help to identify effective policy approaches. However, participation in these initiatives is voluntary. For reasons of national, longitudinal comparability, many countries continue to use their own national surveillance systems for PA and health behavior in children and adolescents, which differ substantially from each other and are difficult to compare.

WHO/Europe and the European Commission are attempting to harmonize PA data collection and policy across the EU through the EU PA Focal Points Network, which was set up following the 2013 Council Recommendation on Promoting Health-enhancing PA (HEPA) across Sectors.^12^ The network is tasked with regularly monitoring PA prevalence and PA promotion indicators within the HEPA monitoring framework for all age groups by analyzing documentation and data collected directly from national governments. This includes the availability of national PA guidelines for children and adolescents, surveillance of PA levels and policies, which mandate PE classes and PA promotion programs in schools.^12^

This study aims to summarize and discuss the ongoing monitoring of HEPA indicators for PA promotion among children and adolescents in the EU by providing a comprehensive overview of results collected through the HEPA monitoring framework. Additionally, the study aims to identify opportunities for future data comparison and analysis by reflecting on the methodology of data collection.

## Methods

Information about the monitoring of PA prevalence, PE and PA promotion for children and adolescents in EU Member States was obtained from the 2018 joint survey conducted by the European Commission and the WHO Regional Office for Europe.^13^ This survey assessed the implementation of the European Council Recommendation on HEPA across Sectors. Data were collected via the EU PA Focal Points Network. The network consists of experts officially nominated by their governments to support data collection on HEPA promotion at the national level. These experts usually work in national ministries of health, ministries of sport, or related national agencies. National HEPA focal points were designated in 2014 by all of the 28 EU Member States at that time following the adoption of the Council Recommendation. Data are collected regularly every three years using a monitoring framework with 23 HEPA-related indicators. Detailed information on definitions, operationalization, and data sources for the indicators can be found in the European Commission’s working document.^14^

For the 2018 round of data collection, focal points from all 28 countries completed an electronic English language questionnaire on behalf of their country that covered all 23 indicators. The questionnaire included five indicators related to children and adolescents’ PA: national recommendations on PA for health (indicator 1), children and adolescents reaching the minimum WHO recommendation on PA for health (indicator 3), PE in primary and secondary schools (indicator 13), schemes for school-related PA promotion (indicator 14) and schemes promoting active travel to school (indicator 16). A list of the questions from the survey used in this study is presented in the Supplementary data. Country focal points answered the questionnaire based on available data from their countries. Responses for each country were extracted for all five indicators. Quantitative and qualitative data from the questionnaires were descriptively analyzed and compared.

## Results

### National recommendations on PA for health

As shown in table 1, 23 out of 28 countries reported providing national PA recommendations for children and adolescents. Predominantly (35.7%), these were based on the 2010 WHO global recommendations on PA. Alternatively, recommendations were based on literature searches or recommendations from other countries, the EU PA Guidelines,^15^ and recommendations from the US Department of Health and Human Services^16^ and the American College of Sports Medicine.^17^ As a result of using various sources, the contents of national PA recommendations for children and adolescents differ slightly between countries: In addition to some differences in the recommendations for duration, intensity and frequency of PA, there are significant variations in age brackets for children and adolescents. Some countries provided PA recommendations for 5–17-year olds along with the WHO, others included 18-year-olds as adolescents. Additionally, some countries had already developed recommendation for under 5-year-old children (for detailed information see also 18).

**Table 1:** Overview on HEPA indicators

Indicator	Number of countries (%)
Indicator 1: National recommendation on PA for health	
Availability for children and adolescents	
Yes	23 (82.1)
No	1 (3.6)
No info	4 (14.3)
Recommendation based on	
WHO	10 (35.7)
Others	6 (21.4)
Combination (WHO + others)	7 (25.0)
No info	5 (17.9)
Indicator 3: Children and adolescents reaching the minimum WHO recommendation on PA for health	
Data availability	
Yes	27 (96.4)
No	1 (3.6)
Age groups	
Children and adolescents separate	19 (67.9)
Children and adolescents together	2 (7.1)
Only adolescents	7 (25.0)
Sex/gender	
Separate	27 (96.4)
Together	0 (0.0)
Unclear/no info	1 (3.6)
Instruments	
Questionnaire	22 (78.6)
Accelerometer	2 (7.1)
Both	4 (14.2)
Cutoff points	
WHO (60 min/day)	24 (85.7)
Others	3 (10.7)
No info	1 (3.6)
Indicator 13: PE in primary and secondary school	
Primary school	
PE lessons	
Hours/times per week	
<3	14 (50.0)
3–6	12 (42.8)
>6	1 (3.6)
No info	1 (3.6)
Mandatory/optional	
All mandatory	21 (75.0)
Mandatory and optional PE classes	3 (10.7)
All optional	3 (10.7)
No info	1 (3.6)
PE quality monitoring	
Yes	19 (67.9)
No	5 (17.8)
No info	4 (14.3)
Secondary school	
PE lessons	
Hours/times per week	
<3	13 (46.4)
3–6	11 (39.3)
>6	1 (3.6)
No info/unclear	3 (10.7)
Mandatory/optional	
All mandatory	21 (75.0)
Mandatory and optional PE classes	4 (14.3)
All optional	2 (7.1)
No info	1 (3.6)
PE quality monitoring	
Yes	20 (71.5)
No	5 (17.8)
No info	3 (10.7)
Involved sectors in the design of the PE curricula^a^	
Education	21 (75.0)
Sports	18 (64.3)
Health	11 (39.3)
Other	5 (17.8)
Indicator 14 and 16: Schemes for school-related PA promotion and for promoting active travel to school^a^	
Active school breaks	16 (57.1)
Active breaks during school lessons	15 (53.6)
After-school HEPA promotion programs	22 (78.6)
Active travel to school	16 (57.1)

a: More than 100% possible due to multiple responses.

HEPA: health-enhancing physical activity; PE: physical education; WHO: World Health Organization.

### Prevalence of PA among children and adolescents

All countries reported conducting surveys to monitor children’s and adolescents’ levels of PA (table 2). Detailed figures on PA prevalence could be retrieved from 27 countries. The total prevalence of children and adolescents reaching recommended levels of PA in EU countries ranged between 6% (Belgium, 6- to 9-year-olds) and 76% (UK/Scotland, 2- to 15-year-olds). Altogether, countries reported PA prevalence data for individuals ranging from three to 18 years of age. Most countries (67.9%) reported PA prevalence separately for children and adolescents. However, the age ranges used to define children and adolescents varied greatly across countries. The cutoff point between children and adolescents ranged between 10 and 13 years. Furthermore, some countries reported prevalence for different ages (e.g. Romania: 11-, 13- and 15-year-olds) while others reported mean values for age groups (e.g. Netherlands: 4- to 11-year-olds and 12- to 17-year-olds). Certain countries (25.0%) only reported PA prevalence for adolescents, which included data from 11- to 18-year-olds (e.g. Austria). All countries that provided data on PA reported prevalence separately for boys and girls.

**Table 2:** Prevalence of PA in in EU member states

Country	Age (years)	Overall (%)	Boys (%)	Girls (%)	Data source	Accelerometer	Cutoff points
Austria	11–17	17.4	23.2	12.5	HBSC—questionnaire	–	WHO
Belgium	6–9	6.0	11.0	2.0	questionnaire	YES	WHO
BE-FR + BE-DE	Overall	15.0			HBSC—questionnaire		
	10–12	23.0	9.0	16.0			
	13–18	14.0	17.0 (at 15 y.)	11.0 (at 15 y.)			
BE-FL	Overall	14.5					
	10–12	17.0	20.6	15.2			
	17–18		14.7	8.2			
Bulgaria	10–19		48.2	25.5	questionnaire	–	WHO
	10–14	32.7	41.8	24.1			
	15–19	39.3	53.1	26.6			
Croatia	11–15	25.7	32.3	19.0	HBSC—questionnaire	–	WHO
	8	88	89	87			
	15	19	25	13			
Cyprus	6–17	–	–	–	IPAQ—questionnaire	–	WHO
	13–17	–	–	–			
Czech Republic	6–17	20.0	30.0	15.0	Questionnaire	–	WHO
	11–15		24.0	18.0			
Denmark	11–15	13.0			HBSC—questionnaire	NO	WHO
	11	16.0	20.0	11.0			
	13	14.0	16.0	12.0			
	15	11.0	15.0	7.0			
Estonia	1–15	16.0	20.0	12.0	Questionnaire	–	WHO
Finland	9	41.0	44.0	39.0	Questionnaire	YES	WHO
	10–11	45.0	50.0	40.0			
	14–15	19.0	23.0	16.0			
	16–17	13.0	16.0	11.0			
	15	17.0	21.0	13.0			
	9–15	31.0	36.0	27.0			
	9–15	34.0			Accelerometer		
	9	51.0	65.0	41.0			
	15	11.0	18.0	8.0			
France	Overall	27.0			Questionnaire	NO	
	3–6	19.0					
	3–10	22.0	25.0	19.0			Number of PA days ≥ 5 and ACS
	11–17	32.0	39.0	26.0			Moderate or intense PA every day or intense PA ≥ 5×/week
Germany	Overall	26.0	29.4	22.4	Questionnaire	YES	WHO
	3–6	45.8	48.9	42.5			
	7–10	26.5	30.0	22.8			
	11–13	19.0	21.4	16.5			
	14–17	11.8	16.0	7.5			
Greece	4–12	59.0	62.2	55.9	HBSC—questionnaire	–	WHO
	13	14.0	19.0	8.0			
	15	11.0	15.0	7.0			
Hungary	11–13–15–17	42.0	50.2	33.8	HBSC—questionnaire	NO	WHO
Ireland	10–12	19.0	27.0	13.0	Questionnaire	NO	EU PA Guidelines
	12–18	12.0	15.0	9.0			
Italy	Overall	56.0			Questionnaire	NO	WHO
	6–10		48.2	51.8			
	8–9	82.0	83.0	81.0			
	11–15	11.0	15.0	8.0			
Lithuania	10–17	9.7	13.6	5.9	Questionnaire	–	WHO
Luxembourg	11–13	24.7			HBSC—questionnaire	NO	WHO
	14–15	19.5					
	16–18	16.1					
	11	28.0	34.0	21.0			
	14	31.0	27.0	34.0			
	18	21.0	26.0	15.0			
Latvia	11–15	19.0	22.0	15.3	Questionnaire	–	WHO
Malta	10–11		28.0	21.0	HBSC—questionnaire	–	WHO
	13		20.0	11.0			
	15		16.0	9.0			
	10–11	25.0	39.0	10.0	Accelerometer		
Netherlands	4–17	44.4 (combination), 46.5 (min/week), 90.2 (bone/ muscle)	46.6 (combination), 49.1 (min/week), 89.7 (bone/muscle)	42.2 (combination), 43.7 (min/week), 90.6 (bone/muscle)	Questionnaire	NO	Combination of 60 min/day and bone and muscle strengthening activities 3×/week.
	4–11	55.5 (combination), 55.5 (min/week), 99.4 (bone/muscle)	56.8 (combination), 56.8 (min/week), 99.3 (bone/muscle)	54.0% (combination), 54.0% (min/week), 99.5 (bone/muscle)			
	12–17	31.0 (combination), 35.5 (min/week), 78.9 (bone/muscle)	34.1 (combination), 39.8 (min/week), 78.0 (bone/muscle)	27.6 (combination), 30.9 (min/week), 79.8 (bone/muscle)			
Poland	11–15	24.2	29.8	18.6	HBSC—questionnaire	NO	WHO
Portugal	Overall		31.0	10.4	Accelerometer	YES	WHO
	10–11	38.0	53.0	23.1			
	14–15	12.0	18.8	5.1			
Romania	Overall (11,13,15)	22.5	29.0	17.0	Questionnaire	NO	WHO
	11		39.0	23.0			
	13		28.0	16.0			
	15		21.0	11.0			
Spain	11–18	24.4	31.7	17.3	Questionnaire	NO	WHO
Slovakia	15–17	10.2	13.4	7.3	Questionnaire	–	–
Slovenia	11	88.0	94.0	81.0	Accelerometer	YES	WHO
	14	69.0	88.0	49.0			
Sweden	11	19.0	23.0	14.0	HBSC—questionnaire	YES	WHO
	15	11.0	13.0	9.0			
UK/England	5–15	22.0	23.0	20.0	Questionnaire	NO	WHO
UK/Northern Ireland	11–16	13.0	17.0	8.0	Questionnaire	NO	WHO
UK/Scotland	2–15	76.0	79.0	72.0	Questionnaire	NO	WHO
UK/Wales	Overall	51.0			Questionnaire	NO	WHO
	3–7	62.0					
	13–17	38.7					

BE-DE+ BE-FR: French and German communities in Belgium; BE-FL: Flemish community in Belgium; HBSC: Health Behaviour in School-aged Children; y.: years; UK: United Kingdom; WHO: World Health Organization; ‘–’: no information available.

While the majority of countries (78.6%) assessed PA via questionnaires, two countries (Portugal and Slovenia) reported PA prevalence based on accelerometer data, and two countries (Malta, Finland) provided both accelerometer and questionnaire data. Regarding the questionnaires, nine countries reported using the questionnaire from the HBSC study,^19^ while the other countries either used nationally developed and validated questionnaires or did not specify, which questionnaires were used. The majority of countries (85.7%) applied the WHO cutoff point (60 min of moderate-to-vigorous PA per day) to determine PA prevalence. France used individualized cutoff points for both children (five or more days of PA per week, and active commuting to school) and adolescents (daily moderate PA or 5 or more days of vigorous PA per week). The Netherlands combined the WHO recommendation of 60 min PA per day with bone and muscle strengthening activities three times per week to indicate whether an individual is sufficiently active or not and to determine PA prevalence among children and adolescents.

### PE in primary and secondary schools

PE is part of the school curriculum in all Member States (table 3). In primary schools, the total number of PE lessons per week range from zero (Luxemburg Grade 1) to seven (Hungary). PE lessons are predominately mandatory (75.0%); in some cases (10.7%), additional PE classes are offered as optional courses. In three countries, however, all PE lessons are optional, and schools get to individually decide how many hours of weekly PE lessons they provide. Whether compulsory or optional, primary school children are offered a total of two to three PE lessons per week, with some differences between age groups. Quality monitoring of PE lessons in primary school was reported in 19 countries, 7 countries reported that the quality of PE lessons is not monitored, and the remaining 4 countries did not provide any information on this issue. Details on strategies and concepts for PE quality monitoring in the single countries are provided in table 3 based on the original answers from the experts.

**Table 3:** PE and monitoring in EU Member States

Country	Hours/times per week (mandatory + optional)		Monitoring
	Primary school	Secondary school	
Austria	Grade 1–2: 2–3 h (optional)	Grade 5–6: 3–4 h (optional)	PE follows a curriculum. In that respect, the quality is monitored. There are no mandatory Output Tests.
	Grade 3–4: 2 h (optional)	Grade 7–8: 3 h (optional)	
		Grade 9–12: 2–3 h (optional)	
Belgium	BE-FR: 2 lessons (50 min)	BE-FR: 2–3 (50 min)	BE-FL: The School Inspection Service organizes general and specific screenings in schools. In 2016–17, a specific screening on the quality of PE was conducted.
	BE-FL: 2 h	BE-FL: 2 h	BE-FR: PE is monitored by the school inspection service. Teachers must apply the recommendations and skills included in the document called ‘Core skills’ for the first stage of secondary schools education. For the rest of the secondary years, teachers must apply the recommendations and skills included in the document entitled ‘Terminal skills and required knowledge’
Bulgaria	3 h	3 h	–
Croatia	Grade 1–3: 3 h	4 year program: 2 h	Primary school: NO
	Grade 4–8: 2 h	3 year program: 1 h	Secondary school: The work of PE teachers is monitored by the Ministry of Science and Education and Education and Teacher Training Agency.
Cyprus	Grade A–D: 1.5 h	Gymnasium: 2–2.5 h	Inspection in school by PE Inspectors
	Grade E and St: 2 h	Grade A: 157,5 min/week,	Primary school: Target Indicators and Target Adequacies are described in detail in the Curriculum of PE in Primary School
		Grades B and C: 135 min/week	Secondary School: Success and Adequacy Indicators are described in detail in the Secondary Education Curriculum of PE
		Lyceum: 1–1.5 h	Random advisory class visits by the school Principal
		Grade A: 67,5 min/week,	
		Grades B and C: 90 min/week	
Czech Republic	2 h + 0–3 h	2 h + 0–4 h (decision of school)	NO, only with Czech school inspection.
Denmark	630 h PE in total per year and 45 min of physical exercise per day		The Ministry of Education oversees the development of 45 min of daily PA through the evaluation and research program in connection to the reform of the primary and lower secondary school. Furthermore, the Ministry follows the consequences of the Reform on the students’ spare time activities e.g. sports and physical activities.
	Grade 1–3: 60 h/year		
	Grade 4–6: 90 h/year		
	Grade 7–9: 60 h/year		
Estonia	2–3 lessons (45 min) School has a possibility to develop it is own curriculum with more PE lessons	In 3 years, 5 courses (one course 35 × 45 min)	There have been several researches to monitor the quality of PE in Estonia—In 2004 and 2008, quantitative research was conducted to map the PE situation, based on the perspectives of PE teachers. In 2013, the physical environment conditions in schools, to organize PE lessons, were mapped. In 2016, qualitative methods (focus group interviews with PE teachers) were used to map the PE situation in Estonia. In addition, several studies were conducted with students to investigate their motivation and attitudes toward PE. In the spring of 2018, Estonian Ministry of Education and Research conducted a large well–being survey among students in 4th, 8th and 11th grade; parents and teachers were also asked about their experiences with PA and PE. This survey continues to be conducted yearly. The majority of this recent research is referred to in the document, ‘PE concept. Upgrading the Estonian PE’ (2017). The purpose of this document was to map the PE situation in Estonia, and to define the reasons for updating PE.
Finland	2–3 lessons (45 min)	2 courses (one 38 h) + 3 courses	A follow-up evaluation on PE learning outcomes, commissioned by the National Agency for Education, was last conducted by the Department of Sports Sciences at the University of Jyväskylä in the spring of 2010.
France	3 h	2–3 h	Primary school: NO
			Secondary school: Inspections from the Education Inspection Offices.
Germany	3 + 6 lessons (45 min, primary schools with a focus on sports and/or PE)	3 lessons (45 min, differences between school types)	Within the framework of quality management in schools.
Greece	3 h	2 h	–
Hungary	5 h + 2 h (sports club activities)	5 h + 2 h (sport club activities)	The national system of teachers’ performance for promotions contains quality aspects regarding PE.
Ireland	1 h	2 h	Primary School: The Primary School PE Curriculum facilitates ongoing assessment of individual children’s abilities and progress by their teachers. PE provision is also monitored by subject inspection, through Whole-School Evaluation (WSE) and/or incidental inspections. PE continues to be included in the program of subject inspections and curriculum evaluations. WSE continue to include a focus on PE in primary schools.
			Secondary school: The Schools Inspectorate is involved in a continuous program of school evaluation; some Inspectors deal specifically with the monitoring and evaluation of PE. Furthermore, as mentioned previously, a number of surveys (HBSC, CSPPA) are ongoing with regard to monitoring PA levels in children.
Italy	2 h	2 h	NO
Lithuania	2–3 h	2–3 h	Primary school: Parametric national indices to be achieved by the pupils are provided in the General Program of PE for Primary School.
			In PE lessons for primary school, criterion based assessments of pupils’ skills and knowledge is applied; these assessments are based on the individual progress (idiographic) approach instead of the measurable result. Fitness tests can also be applied to evaluate individual fitness changes and support better recognition of individual strengths and weaknesses. The main criterion for the evaluation of a teacher’s work is considered to be individual progress of a child together with developed need to be physically active.
			Secondary school: Quality of PE is monitored in accordance with the general state system of Education Monitoring, and executed by the National Agency for School Evaluation. Evaluation is performed every 7 years.
Luxembourg	Grade 1: 0 h	First year: 3 h	–
	Grade 2–5: 2 h	Next 5 years: 2 h	
	Grade 6–7: 2 h	Last 2–3 years: 2 h	
Latvia	2 h	3 h	Although there is no specific monitoring system for PE in place, there is a normative regulation on the state standard in basic education, the subjects of study standards in basic education and model basic educational programs.
			Both normative set the evaluation standards and principles. Besides that every educational institution has developed the internal regulation which defines the evaluation of the learning process and procedure including PE. The quality of PE is taken into account during the accreditation process.
Malta	2 h (optional)	2 h (optional)	–
Netherlands	2 h (optional)	2–2.5 lessons (50 min)	The Dutch Inspectorate of Education is responsible for the inspection and review of schools and educational institutions. In 2018, a report was published about characteristics and trends in PE in primary schools.
	Grade 1–2: average of 113 min		
	Grade 3–8: average of 89 min		
Poland	Grade 1–3: 3 h	3 h	In accordance with polish law, PE teachers are supervised by school headmasters who observe their work on a daily basis (internal pedagogical supervision). Headmasters are supervised by regional education authority/board -Kuratorium oświaty (external pedagogical supervision).
	Grade 4–8: 4 h		
Portugal	3 h	2.25 h	The curriculum is being monitored by the Ministry of Education. The Ministry of Education has its own inspection system to monitor if the PE program is being addressed by teachers.
Romania	Grade: 1–2: 2 h PE and 1 h play and movement + 1 h play and movement	Grade 5–7: 2 h + 1 h	The quality of PE is monitored through inspections.
	Grade 3–4: 2 h PE and 1 h play and movement	Grade 8: 1 h + 1 h	
Spain	2 h	2 h	In different regions, they are monitoring the physical condition of the students (within the PE classes).
Slovakia	3 h	3 h	NO
Slovenia	2–3 lessons (45 min)	1–3 lessons (45 min)	NO
Sweden	Grade 1–3: 1.5 h and 30 min daily PA	1–1.5 h (100 h over 3 years) + 1 optional course (100 h)	The Swedish Schools Inspectorate performs quality audits, on a non-regular basis, on various quality aspects of schools and education. Each quality audit covers a small selection of schools only. Recent quality audits concerning some aspects of PE have been conducted in 2010 and 2012. There is also an ongoing audit covering 20 schools. No audit concerning PE in upper secondary education has been conducted in recent years.
	Grade 4–9: 2 h and 30 min daily PA		
UK/England	2 h (optional)	2 h (optional)	NO
UK/Northern Ireland	2 h (optional)	2 h (optional, up to schools to decide how much time to allocate to PE)	The Education and Training Inspectorate is responsible for inspecting the quality of provision across all areas of the statutory curriculum, including PE.
UK/Scotland	2 h	2 h	Primary school: Standards measured as part of school reporting framework.
			Secondary school: Her Majesty's Inspectors of Education and Education Scotland periodically inspect all schools and produce reports.
UK/Wales	2 h	2 h	NO

Note: all information are based on expert data from the HEPA indicators survey, ‘–’: no information available.

In secondary schools, between one (e.g. Croatia, Romania) and seven (Hungary) PE lessons are provided weekly. In most countries (75.0%), PE lessons are mandatory. Four countries additionally offer optional PE classes within their PE curriculum. The quality of PE in secondary schools is monitored within 20 countries (table 3).

Across EU Member States, different sectors are involved in the design of PE curricula. In addition to the education sector, the sport sector is involved in most countries. Eleven EU Member States reported that the health sector also participated in curriculum design. Other parties concerned are sometimes also involved, including for example teachers or science and education providers.

### National schemes for PA promotion

Beyond PE classes, the most commonly used national schemes for school-related PA promotion are after-school HEPA promotion programs (table 4). To facilitate these programs, schools either cooperate with local organizations or independently provide voluntary after-school sports activities. In more than half of the EU Member States, national schemes exist for active school breaks, active travel to school, and active breaks during school lessons. Some countries provide multicomponent interventions in schools, which address different opportunities for PA during the school day. Examples include the ‘Active School Flag’ in Ireland (www.activeschoolflag.ie), the ‘School in Motion’ project in Estonia (www.liikumakutsuvkool.ee), or the ‘Finnish Schools on the Move’ program (www.liikkuvakoulu.fi). Most active travel schemes seek to promote cycling to school (e.g. Croatia, Czech Republic, Denmark, Hungary, Slovak Republic) and include cooperative work with stakeholders from the transport sector to improve cycling infrastructure. One example for a national action plan is the ‘Octopusplan’ in Belgium (www.octopusplan.info) which promotes sustainable active travel to and from school by building up child-friendly school environments.

**Table 4:** PA promotion in EU member states

Country	Active school breaks	Active breaks during school lessons	After-school HEPA promotion programs	Active travel to school
Austria		✓	✓	✓
Belgium	✓	✓	✓	✓
Bulgaria	✓		✓	
Croatia	✓	✓	✓	✓
Cyprus	✓	✓	✓	
Czech Republic	✓		✓	✓
Denmark	✓	✓	✓	✓
Estonia	✓	✓		✓
Finland	✓	✓	✓	✓
France			✓	✓
Germany	✓	✓	✓	
Greece			✓	
Hungary			✓	✓
Ireland	✓	✓	✓	✓
Italy	✓	✓	✓	✓
Latvia		✓		
Lithuania	✓		✓	
Luxembourg		✓	✓	✓
Malta			✓	
Netherlands				
Poland				
Portugal			✓	
Romania	✓	✓	✓	✓
Slovakia				✓
Slovenia	✓		✓	
Spain		✓		
Sweden	✓		✓	✓
UK	✓	✓	✓	✓
Total	16	15	22	16

## Discussion

This study summarizes the current monitoring of HEPA indicators for PA promotion among children and adolescents in all EU Member States providing a comprehensive assessment of where the EU stands in regard to PA promotion and monitoring for this population group. The five available HEPA indicators related to children and adolescents cover important aspects of PA promotion policy: national PA recommendations, PA prevalence, PE policies, PA promotion in schools and active travel.

In general, the EU PA Focal Points Network facilitates the provision of synchronized and comparable data across Member States, as information is gathered simultaneously by government representatives. The national focal points are usually closer to policymakers in their countries than to research; however, network meetings always include exchange with experts from scientific society about the latest developments in the field. Such an approach allows to shorten a gap between research and policy. This successful cross-national, government-driven policy monitoring may encourage replication in other world regions. However, our analysis of the responses provided by the countries showed that, despite the large number of European initiatives to promote PA for children, HEPA policies differ between countries and still have room for improvement. In some cases, a more critical reflection on the indicators used and on potential ways of improving the quality of the available data might be warranted.

### Indicator ‘national recommendations’

Most EU countries have national PA recommendations for children and adolescents in place. The majority is in line with WHO recommendations, but there are some disparities in duration, intensity, and frequency of PA, and significant variations in chosen age ranges.^18^ However, there remains a need for further action, as some countries have not yet developed recommendations and discrepancies remain between countries that have them. In addition, EU governments might benefit from greater synergies by collaborating more closely when developing and updating future PA recommendations.

While assessing the availability of PA recommendations in EU Member States is a step toward harmonizing PA monitoring, a further step might be a shift from identifying the ‘existence’ of recommendations to assessing their ‘quality’. An adapted indicator should presumably assess if national recommendations are up-to-date, whether and how closely they resemble the WHO recommendations (e.g. in regards to age brackets), if they cover all individuals of different ages and/or physical conditions (e.g. chronic diseases) and contain all relevant components (e.g. amount, frequency, activity types (e.g. aerobic PA, muscle-strengthening activity), duration, restrictions (e.g. for people with NCDs)), if they were drawn up using the latest available evidence, and if their development was accompanied by a national capacity building process or suitable dissemination efforts.^20^

### Indicator ‘prevalence of PA among children and adolescents’

In line with previously published international studies on PA prevalence,^6^^,^^21^ we identified low levels of PA in children and adolescents among boys and girls in nearly all EU Member States. In comparison to other international data sources (e.g. 2017/2018 HBSC survey), our data showed a greater variance of PA prevalence between countries.^7^^,^^21^ In the 2017/2018 HBSC survey on PA prevalence in 11-, 13- and 15-year-olds in Europe, between 4% (15-year-old girls in Italy) and 52% (11-year-old boys in Finland) adolescents reported at least 60 min of moderate-to-vigorous PA daily.^21^ In line with our results, in all age groups, boys were more physically active than girls.^21^

Moreover, in the Global Matrix 3.0 PA report card for children and youth, PA prevalence and action was rated in 20 European countries.^7^ Similar to our results, prevalence differed between countries ranging from grade A- (80–86% of children and youth meeting the WHO recommendation for PA) in Slovenia to grade F in Belgium and Scotland (≤20% of children and youth). In general, countries received ratings between C and D (20–53% of children and youth), which corresponds to our reported PA prevalence in EU Member States.

Although 27 out of 28 EU Member States provided prevalence of PA in children and adolescents, comparing prevalence across EU Member States (based on the data from the EU PA Focal Points Network) is still difficult; this is due to the fact that reporting methods varied and different instruments (e.g. self-reports, accelerometer) were used. Although the majority of countries used questionnaires, a comparison is still not applicable as some used standardized, reliable, and validated questionnaires assessing different domains of PA (e.g. HBSC, IPAQ) while others used single item questions or did not further specify how PA was assessed. Additionally, countries applied different cutoff points to estimate if a child is active or inactive. In order to improve comparability of PA prevalence, EU Member States would benefit from applying the same recommendations and cutoff points and by using the same instruments, preferably including the measurement of objective accelerometer data.

### Indicator ‘PE in primary and secondary schools’

Schools are an important setting for PA promotion, as children in EU Member States are obliged to attend them and spend a significant part of their day there.^22^ With regard to PE in primary and secondary schools, our results revealed that PE lessons are predominantly mandatory in the school curriculum of EU Member States; however, there are still differences between, as well as within, countries regarding the number of PE lessons per week and their duration. The total number of hours of PE is often defined differently by Member States due to different ways of reporting PE. Some countries reported PE lessons per week including the duration (in min) of one PE lesson, others reported hours of PE per week, and the remaining countries reported PE in hours per year.

A unified way of reporting PE would allow for better comparability of data between countries. Especially in the school setting, different administrative levels (e.g. national, state) and sectors (e.g. health, education) play a role, which may complicate data collection and comparison efforts across countries.

Many countries reported that several sectors were involved in the development of the PE curriculum. However, we also found that, in 25% of Member States, the implementation of the curriculum and the quality of PE lessons were not monitored. This would be an important addition as the quality of PE might directly influence PA behavior in children and adolescents. Currently, no comparison of monitoring and quality of PE lessons between EU Member States is possible as concepts and methods for monitoring differed greatly between countries and no consistent definition of quality underlies the responses from the experts. Consequently, using a standardized monitoring and surveillance system with specified quality indicators for PE lessons, for example the one provided by UNESCO,^23^ might enhance the effectiveness of PA promotion monitoring in EU Member States. In this context, the nature and aims of PE need to be considered: A growing body of research addresses a shift from being physically active in PE lessons to an empowerment and motivation for a lifelong health-related participation in PA. This concept of physical literacy is based on a multidimensional approach that incorporates physical capabilities and affective, cognitive, and psychosocial aspects of exercise and sport in a holistic understanding.^24^

### Indicator ‘national schemes for PA promotion’

Beyond PE lessons, further PA promotion in schools is crucial to help children and adolescents reach PA recommendations. This article provides an overview of national schemes for school-related PA and active travel to school in EU Member States. Almost all EU Member States reported at least one PA promotion program. However, schemes for active school breaks, active school breaks during lessons and active travel to school exist in only around a half of EU Member States. In the survey, experts were asked to provide data on the availability of PA promotion programs. However, no information was provided on the reach, efficacy, scale and sustainability of the interventions that were named. A further comparison of existing schemes in terms of feasibility, reachability of children and adolescents with diverse socio-demographic and cultural backgrounds, and longitudinal effects on children’s and adolescents’ PA is recommended to foster PA promotion program implementation and to enable other countries to adopt effective schemes. Future research would benefit from examining success stories in more detail to determine what lessons can be learned and applied to other countries.

### Limitations

Although this study is, to the best of our knowledge, unique in presenting data on PA prevalence monitoring and PE and PA promotion for children and adolescents among all EU Member States from a policy perspective, it has some limitations. First, all answers were based on expert responses from national governments. Thus, the study is limited to qualitative answers from surveys, and no direct information is available on what EU Member States actually do. Second, problems sometimes occurred with the interpretation of the answers. For example, the answers ‘no’ or ‘–’ may have meant that the focal point is certain that no data/program exists for a certain item or, alternatively, that the focal point had no information/access on existing programs/data.

Furthermore, this study describes the situation in 2018. The COVID-19 crisis in 2020/21 has come with new challenges for PA promotion and PE.^25^ Safe and accessible PA promotion strategies need to be developed to reach vulnerable groups, which suffer predominantly from negative effects of COVID-19 crisis such as children with psychological or developmental problems or socially disadvantaged children. Still, the information on the status of PA promotion before the COVID-19 crisis can help countries to combine their efforts in finding new long-term solutions in the future.

## Conclusions

In general, it can be said that the use of the EU PA Focal Points Network for monitoring HEPA indicators enables the simultaneous collection of harmonized data in all EU Member States. All data are directly obtained from governments, which makes it possible to study PA promotion policy initiatives in these regions. Regular data collection makes it possible to track progress on each of the indicators. At the same time, despite the unified approach to monitoring HEPA indicators, it is still difficult to make comparisons between countries. The information is provided in different formats, and its quality varies between countries. Publishing the results of the HEPA monitoring framework in the form of country factsheets^13^ and scientific articles (e.g. Breda et al.,^9^ Gelius et al.,^18^ Whiting et al.^26^) can raise awareness among countries about the overall situation in the region and facilitate the sharing and adoption of best practices.

Our findings also let us conclude that the current EU monitoring system on PA promotion among children and adolescents should be adapted to provide evidence that can inform future policy development. Specifically, we recommend the following changes:

1. For some indicators (e.g. national recommendations), a shift from assessing quantity (‘Do they exist—yes/no?’) to quality (‘What are they?’) seems advisable.
2. In general, the EU should try to find ways to shift from national assessment instruments toward harmonized assessment, which allows for better comparison between Member States while preserving the assessment of trends over time.
3. It might also be necessary to consider a partial shift from a ‘stable’ assessment (i.e. always using the same indicators) to a more flexible and agile system that allows updating and changing indicators if they are no longer relevant.

## Supplementary data

Supplementary data are available at *EURPUB* online.

## Funding

No funding.

*Conflict of interest*: None declared.

## Disclaimer

The writing group takes sole responsibility for the content of this article, and the content of this article reflects the views of the authors only. KW and SW are staff members of the WHO. The authors alone are responsible for the views expressed in this publication, and they do not necessarily represent the decisions or the stated policy of the WHO.

## Data availability

The data underlying this article will be shared on reasonable, written request to the WHO Regional Office for Europe, but written consent of the PA Focal Points of involved countries, the European Commission and the WHO Regional Office for Europe may be required.

Key points

- The EU PA Focal Points Network for monitoring HEPA indicators enables the simultaneous collection of harmonized data on PA prevalence and promotion in children and adolescents in all EU Member States.
- 82% of EU Member States developed national recommendations on PA, 96% provided data on prevalence of children and adolescents reaching the minimum country-specific recommendation on PA.
- After-school HEPA promotion programs were mostly implemented in EU Member States (78.6%), followed by active school breaks (57.1%), active travel to school (57.1%) and active breaks during school lessons (53.6%).
- The findings of this study show that current EU monitoring system on PA promotion among children and adolescents should be adapted to provide evidence that can inform future policy development.

## Supplementary Material

ckab193_Supplementary_Data
