# Supplementary material for: Monitoring of physical activity promotion in children and adolescents in the EU: current status and future perspectives
Source: Eur J Public Health. 2021 Nov 17;32(1):95–104. doi: 10.1093/eurpub/ckab193 (PMC8807108; doi:10.1093/eurpub/ckab193)
Supplement: ckab193_Supplementary_Data [file ckab193_supplementary_data.docx]

**Supplementary Material: Overview on HEPA Monitoring Framework**

| **Indicator 1: National recommendation on physical activity for health** | | |
| --- | --- | --- |
| Does a national recommendation on physical activity and health exist in your country, i.e. an officially adopted statement on the duration, intensity and frequency of physical activity behaviour that the population should reach? | | |
| If YES, specify for which age groups: For children and youth (5-17) | | |
| National recommendation are based on | | |
| WHO recommendations (2010) | | |
| Other? (Specify) | | |
| **Indicator 3: Children and adolescents reaching the minimum WHO recommendation on physical activity for health** | | |
| What is the total prevalence of children and adolescents reaching the recommended levels of physical activity in your country? (%) | | |
| Which is the cut-off point used? | WHO (60min per day)? | |
|  | Other? (Specify) | |
| Are children and adolescents reported together or separately? | | |
| If together, specify: | Age range used (*x* to *y* years) | |
|  | Prevalence for boys (%) | |
|  | Prevalence for girls (%) | |
| If children and adolescents are reported separately, specify: | | |
| For children: | Prevalence (%) | |
|  | Age range used (*x* to *y* years) | |
|  | Prevalence for boys (%) | |
|  | Prevalence for girls (%) | |
| For adolescents | Prevalence (%) | |
|  | Age range used (*x* to *y* years) | |
|  | Prevalence for boys (%) | |
|  | Prevalence for girls (%) | |
| Which instrument was used? | Questionnaire | |
|  | If YES, specify which questionnaire: | |
|  | Accelerometers | |
|  | Other? (specify) | |
| **Indicator 13: Physical education in primary and secondary school** | | |
| **Primary School** | | |
| What is the total number of hours of physical education per week provided in primary schools? | | |
| All mandatory? | | |
| If NO, specify how many hours are mandatory? | | |
| Is the quality of physical education being monitored? (e.g., benchmarks, UNESCO checklist) | | |
| If YES, provide details | | |
| **Secondary School** | | |
| What is the total number of hours of physical education per week provided in secondary schools? | | |
| All mandatory? | | |
| If NO, specify how many hours are mandatory? | | |
| Is the quality of physical education being monitored? (e.g., adherence to benchmarks, inspections) | | |
| If YES, provide details | |  |
| Which sectors were involved in the design of the PE curricula? | | |
| Education | |  |
| Sports | |  |
| Health | |  |
| Other? (specify) | |  |
| **Indicator 14 and 16: Schemes for school-related physical activity promotion and for promoting active travel to school** | | |
| Does your country have a national scheme for active school breaks (i.e. breaks between school lessons)? | | |
| If YES, provide details | | |
| Does your country have a national scheme for active breaks during school lessons? | | |
| If YES, provide details | | |
| Does your country have a national scheme for after-school HEPA promotion programmes (at schools, at sport clubs or in communities)? | | |
| If YES, provide details | | |
| Does a national scheme exist to promote active travel to school (e.g. walking buses, cycling)? | | |
| If YES, provide details | | |
